# Age-associated Cognitive Decline: Insights into Molecular Switches and Recovery Avenues

**DOI:** 10.14336/AD.2015.1004

**Published:** 2016-03-15

**Authors:** Arpita Konar, Padmanabh Singh, Mahendra K. Thakur

**Affiliations:** 1 Department of Zoology, Banaras Hindu University, Varanasi 221005, India; 2 CSIR-Institute of Genomics and Integrative Biology, New Delhi 110025, India

**Keywords:** cognitive decline, brain aging, gene expression, molecular mechanism, recovery

## Abstract

Age-associated cognitive decline is an inevitable phenomenon that predisposes individuals for neurological and psychiatric disorders eventually affecting the quality of life. Scientists have endeavored to identify the key molecular switches that drive cognitive decline with advancing age. These newly identified molecules are then targeted as recovery of cognitive aging and related disorders. Cognitive decline during aging is multi-factorial and amongst several factors influencing this trajectory, gene expression changes are pivotal. Identifying these genes would elucidate the neurobiological underpinnings as well as offer clues that make certain individuals resilient to withstand the inevitable age-related deteriorations. Our laboratory has focused on this aspect and investigated a wide spectrum of genes involved in crucial brain functions that attribute to senescence induced cognitive deficits. We have recently identified master switches in the epigenome regulating gene expression alteration during brain aging. Interestingly, these factors when manipulated by chemical or genetic strategies successfully reverse the age-related cognitive impairments. In the present article, we review findings from our laboratory and others combined with supporting literary evidences on molecular switches of brain aging and their potential as recovery targets.

Cognitive decline is a major hurdle for the attainment of healthy aging. Advancing age impacts our cognitive capacities of perceptual speed, attention, reasoning, and the most crucial, learning and memory processes dependent on hippocampus and frontal cortex [1]. Mild impairment in these brain functions is manifested even in physically healthy aged individuals and its severity disposes to neurodegenerative disorders. The demographic survey worldwide estimates that 40% of population above 60 years is affected by varying degrees of cognitive decline, which is persistently increasing in prevalence and transition to disorders [2]. In this regard, major research efforts focus towards understanding the pathological aging such as Alzheimer’s and Parkinson’s disease. It is worth mentioning here that even if these disorders are devastating, all the aged people are not afflicted with dementia. However, majority of individuals suffer from subtle age-associated cognitive deficits which deteriorate quality of life. Therefore, unraveling the mechanisms of cognitive decline associated with normal aging is of utmost importance and would offer a hope for recovery targets of degenerative pathologies.

The molecular mechanisms underlying the onset of age-associated deteriorations in brain is still a mystery and one of the greatest health challenges of the decade. However, every cloud has a silver lining as certain examples of healthy aged brain raised immense hope among the researchers. The neuroscientists from University of Groningen, Netherlands assessed the cognitive abilities of 113-year old Dutch woman, Hendrikje van Andel-Schipper, and examined her brain immediately after the death at the age of 115. Her cognitive performances were better than healthy adults of 60-75 years. Her brain was without plaques, vascular changes and neuron number corresponded with brains of healthy people of 60-80 years [3].

These observations have made researchers to believe that there are no limits to healthy life of brain and challenged them to search the measures which maintain cognitively intact brain till late age. In this context, the major questions that strike the neuroscientists are why some aged individuals can preserve cognitive abilities while others can’t and which factors switch normal or non-pathological brain aging to disease conditions.

## Gene expression changes in aging brain: Central to cognitive decline

The triggers and consequences of brain aging and associated cognitive decline are considered multidimensional, though accumulating studies implicate that gene expression changes are pivotal [4]. Alterations in gene expression robustly affect brain functions from cellular to behavioral level with critical influence on cognitive processes. Although major investigations have been conducted for gene expression changes in neurodegenerative disorders, little is known about their profiles in aged brain. Moreover, studies conducted till date reveal that genes related to stress, inflammation, immune response, mitochondrial functions, growth factors, neuronal survival and calcium homeostasis are altered during aging [5,6]. In general, genes that are stress responsive and related to inflammation and DNA repair are upregulated while genes involved in neuronal growth and survival and mitochondrial functions are downregulated with advancing age in several organisms [7]. Gene expression changes during brain aging also exhibit regional differences and sexual dimorphism [8]. The forebrain regions including superior frontal gyrus, entire cortex and hippocampal CA1 are more susceptible to aging and exhibit a large number of gene alterations [9].

Studies from our laboratory have primarily focused on specific categories of genes influencing cognitive functions during aging and also implicated in neurodegenerative disorders. As hormonal changes are also crucial during aging process, we have initially examined steroid hormones and their receptor encoding genes in brain aging. In parallel, we studied causative and susceptibility genes of neurodegenerative disorders in normal brain aging process and checked their regulation by the steroids. These studies revealed very promising results and prompted us to believe that cognitive impairment during aging also involves some other factors. Further literature evidences indicated the concept of synaptic plasticity decline in brain aging. In the past few years, age-associated cognitive decline has been primarily attributed to loss of synaptic connectivity and plasticity rather than complete degeneration of neurons. Therefore, we started investigating genes involved in different stages of synaptic plasticity and their influence on cognition, particularly learning and memory in aging brain.

## Sex steroids and receptors

An important factor influencing cognitive functions of aging brain is hormone. In particular, alteration in the level of sex steroid hormones including testosterone, estrogen and progesterone [10] is considered a serious risk factor for accelerated brain aging. Amongst these steroids, estrogen stands out for its direct influence on cognitive decline. The estrogen deprivation in postmenopausal women is integral to aberrant changes in hippocampal morphology and ensuing cognitive impairment. Estrogen acts mainly through its intracellular receptors, estrogen receptor (ER) α and β. These receptors are implicated in several crucial brain functions including neuronal growth and differentiation, migration and synapse formation [11, 12].

The expression of these receptors encoding genes is significantly reduced in aging brain that is influenced by estrogen, growth, thyroid and other hormones [13]. Cumulative evidences link the decrease of ERs with enhanced vulnerability for cognitive deficits in aging as well as neurodegenerative disorders. A linear relationship was found between cognitive function and level of ERα in the frontal cortex of Alzheimer's disease (AD) patients [14]. In rodent models, aged females showed lower ER α and β immunoreactivity consistent with reduction in the number of hippocampal synapses [15].

ER α and β following ligand binding recruit a set of coactivators and corepressor proteins termed as coregulators which regulate expression of specific genes. Interestingly, the expression of these coregulators and their interaction with ERs also alter in aging brain. As compared to young, old mice exhibited significant decrease in the level ERα coregulators such as PELP1, RIP140, PGC1a and BAF60. PELP1 and RIP140 are key players of estrogen-induced neurogenesis and learning and memory, whereas p68 and PGC1α have been implicated in formation and maintenance of dendritic spine and neuronal growth. These data provided evidence that age dependent decline in ERα coregulators might be responsible for cognitive dysfunctions [16]. ER β coregulators AIB1, ERAP 140, TrkA, Src, pCREB and CREB also decreased in aging brain and eventually influenced estrogen signaling. CREB is central to learning and memory regulating a wide variety of intracellular signaling cascades responsible for synaptic efficacy and long-lasting changes in synaptic plasticity. CREB is phosphorylated (pCREB) in the nucleus by various protein kinases via secondary messengers such as cAMP and/or Ca^2+^ for regulating specific genes. Alterations in CREB signaling lead to cognitive deficits in aging and degenerative pathologies [17]. These compelling evidences have put forward the use of estrogen replacement therapy in reversal of cognitive deficits. However, estrogen replacement therapy in post-menopausal women may be a risk factor for development of breast cancer. To overcome this problem, selective estrogen receptor modulators (SERM) may play a promising role due to its selective action as an agonist and antagonist in tissue dependent manner. Recently, Kindler et al [18] reported that raloxifene, a benzothiopene derivative SERM improved hippocampal activity and associated learning in schizophrenia patients. Earlier reports have shown that raloxifene treatment prevented osteoporosis and reduced the risk of breast cancer in post-menopausal women. Thus, selective modulation of estrogen receptors and coregulators may be identified as potential therapeutics in aging [19, 20, 21].

## AD associated genes

The severity in aging associated cognitive decline predisposes to neurodegenerative diseases such as AD. Although majority of patients develop late-onset AD at an age above 65 years, 2-10% of patients show clinical symptoms much earlier referred to as early-onset AD. Therefore, cognitive aging is not causative rather predisposing factor making individuals vulnerable for AD. Age related structural and functional deteriorations in brain are considered to be the greatest risk factor for late onset AD. Mutations in different genes including amyloid precursor protein (APP), presenilin (PS) 1 and 2 are considered to cause familial forms of AD whereas apolipoprotein E (ApoE) 4 allele increases the susceptibility. We hypothesized that these candidate genes that pose risk for AD might also alter during normal aging. Expression analysis of these AD related genes during aging in vulnerable brain regions of cerebral cortex and hippocampus confirmed our speculation. Moreover, we also noted pronounced influence of estradiol and testosterone on the expression of these genes in aging brain.

AD is characterized by accumulation of amyloid beta peptides produced by proteolytic cleavage of APP. APP gene expression showed significant upregulation in the cerebral cortex of old male and female mice. Further, estradiol modulated the expression APP mRNA as well its alternative splicing in old mouse cerebral cortex. APP has various isoforms (APP770, APP751 and APP695) amongst which APP695 is predominantly found in the brain and remains elevated in non-pathological conditions. APP695 was significantly upregulated by estrogen in aged female mouse brain [22].

Other genes associated with early onset of AD are PS1 and PS2, which are transmembrane aspartyl proteases. They constitute the catalytic core of the gamma-secretase enzyme which cleaves βAPP to generate Aβ peptides. We observed reduction in PS1 expression but increase in PS2 in the cerebral cortex of old mice of both sexes. Such expression pattern was modulated by 17β-estradiol and testosterone [23, 24].

Interestingly, all the AD associated genes were not affected during aging as we noted no significant change in the expression of ApoE associated with late-onset sporadic and familial AD. ApoE is a lipid transport protein involved in membrane repair and synaptic plasticity and implicated in atherosclerosis and neurodegeneration [25, 26, 27], but its overall impact during normal aging remains to be determined. We reported that ApoE expression decreased from young to adult and then remained unchanged in old mouse cerebral cortex. Certainly, such expression profile suggested the involvement of other factors in maintaining ApoE level and dependent brain functions in old [28].

## Synaptic plasticity genes

Over the past decade, enormous evidences from human and animal models suggest that brain aging accompanied cognitive decline is primarily due to changes in neuronal morphology and synaptic plasticity. Synaptic plasticity events form the core of learning and memory processes that are affected almost in every species during aging. Aging rodents show reduced hippocampal dependent recognition memory [29], spatial memory, fear memory [30] and associative memory. Similarly, primates and non-primate mammals show decline in working memory [31].

Memory processes dependent on hippocampus (declarative memory) and dorsolateral prefrontal cortex (working memory) show maximum decline with advancing age [32, 33, 34]. In the light of this information, we were intrigued to investigate the genes regulating synaptic plasticity in aging brain. Neuronal activity induced genes such as brain derived neurotrophic factor (BDNF) and activity regulated cytoskeleton associated protein (Arc) have been well reported in synaptic plasticity changes of aged brain [35]. We explored a new class of molecule, activity dependent neural proteases in brain aging. Upon neuronal stimulation, these proteases are secreted in the synaptic cleft and cleave extracellular matrix proteins and/or cell adhesion molecules. This proteolytic cleavage immediately causes synaptic remodeling or the cleaved products activate certain signaling cascades, eventually affecting synaptic connectivity and plasticity.

Neuropsin (NP) is one such activity induced serine protease predominantly expressed in hippocampus CA1-CA3 subregions, amygdala and moderately in prefrontal cortex. NP gene ablation impaired long-term potentiation (LTP) and memory acquisition process [36, 37, 38]. NP cleaves L1CAM, an immunoglobulin superfamily neural cell adhesion molecule, and is implicated in hippocampal plasticity [39]. L1CAM is also known to regulate neurite growth of hippocampal neurons by inducing the expression of microtubule associated protein (MAP)2c [40]. With this background, we investigated NP and its effectors L1CAM and MAP2c in aging mouse brain. We observed age and region dependent variation in NP expression. NP mRNA and protein expression was drastically decreased in the olfactory bulb, cerebral cortex and hippocampus of old mice as compared to young. In cerebral cortex, the decline was gradual with age while in olfactory bulb and hippocampus there was a robust increase in adult and decrease in old. As these brain regions are pivotal for memory processes, it indicated that NP might be a critical determinant of regional differences in memory decline during aging. Analysis of L1CAM cleavage and MAP2c protein level revealed regional and age dependent strong positive correlation with NP expression pattern. These findings suggested that NP dependent LICAM cleavage might regulate neurite growth marker MAP2c attributing to alterations in synaptic plasticity and memory during aging [41].

Neurite growth inhibitory genes implicated in synaptic plasticity were also altered during aging. Nogo-A is a myelin-associated neurite growth inhibitory protein, that binds to its receptor Nogo-66 receptor1 (NgR1) and regulates filamentous actin dynamics and involved in structural plasticity of the synapses. We demonstrated that Nogo-A protein level decreased in male and female forebrain of old mice while NgR1 remained unaltered [42]. In recent years, another set of synaptic proteins neurexins (Nrxns) and neuroligins (Nlgns) have emerged as potential candidates for alterations in structural synaptic plasticity. Presynaptic Nrxns form complexes with postsynaptic Nlgns [43] and facilitate the differentiation, maturation, and stabilization of synapses [44, 45]. We analyzed expression pattern of these genes in aging mouse brain and observed a significant decrease of both Nrxn1 and Nlgn3 level in cerebral cortex of old mice. Interestingly, this pattern strongly correlated with a crucial marker of functional synapse, synaptophysin during aging [46].

## Master regulators of gene expression: Epigenetic switch?

The molecular switches driving these wide spectrums of gene expression alteration in aged brain deserve utmost attention. Research endeavors over past few years hypothesize that epigenetic mechanisms of DNA methylation and post translational modifications of histones modulate gene transcription resulting in age-related cognitive deficits. In particular, methylation of DNA and histone acetylation is considered pivotal for memory-linked gene transcription [47].

DNA methylation occurs at 5′C of cytosine in CpG dinucleotide and is distributed as CpG islands at the promoter region of gene and on repetitive sequences in the genome. DNA methylation directly interferes with the binding of transcription factors or recruits methyl binding proteins (MBDs and MeCP2) forming repressor complex at the gene promoter and thereby down regulates gene expression. Global methyl cytosine level is decreased while promoter specific methylation either gets decreased or increased in the brain during aging. Such alteration in cytosine methylation patterns affects synaptic plasticity gene expression and function, particularly learning and memory during aging. Further, alteration in DNA methylation is one of the key factors leading to neurodegeneration and cognitive dysfunction in AD and other neurological disorders. AD human subjects showed decline in DNA methylation level in CA1, CA3 and dentate gyrus region of hippocampus as compared to age matched controls [48, 49, 50, 51].

Although there is reduction in global level of DNA methylation during aging or AD, methylation at gene promoter level is different. It is observed that DNA methylation at the promoter region of Arc is upregulated in the hippocampus of old mice as compared to adult [52]. This upregulation in DNA methylation is associated with decline in Arc expression and memory consolidation in old mice. BDNF, a well known synaptic plasticity gene and marker for learning and memory, plays an important role in neuronal survival and synaptic activity. DNA methylation at the promoter region of BDNF is upregulated in the peripheral blood sample of AD patients as compared to age matched control [53]. Moreover, APP gene upregulation causing Aβ deposition in brain of AD patients has been attributed to hypomethylation at gene promoter [54, 55]. DNA methylation is catalyzed by DNA methyl transferases (DNMTs) categorized into DNMT1, 3a and 3b. DNMT3a and 3b are de novo methyl transferases that methylate previously unmethylated sites while DNMT1 is maintenance methyltransferase methylating hemi methylated sites on DNA strands. Earlier reports have shown that DNMTs play a crucial role in brain development, neuronal differentiation, apoptosis and cognition. We reported that DNMT1 is down regulated in the cerebral cortex and hippocampus and positively correlated with decline in recognition memory consolidation during aging [56]. In consistent with our findings, cumulative studies on DNMT1 established its crucial role in memory processes. Homozygous DNMT1 mutation caused lethality at embryonic stages [57] while conditional DNMT1 mutation in rodents led to cortical and hippocampal neurodegeneration, and reduced their volume and memory consolidation [58, 59, 60] DNMT1 mutation in human caused hereditary sensory neuropathy characterized by reduced cytosine DNA methylation level, hearing loss, neurodegeneration and dementia [61].

The post translational modifications of histones remodeling structure of chromatin are also considered significant for gene expression changes in aging brain. Early in eighties, we reported for the first time that histone acetylation [62], phosphorylation [63] and methylation [64] levels declined in the brain of aging rats. The decline in histone acetylation level was positively correlated with in vitro mRNA synthesis during aging. Several studies thereafter highlighted the role of histone acetylation in cognitive decline during aging and disorders [30, 65, 66]. Further it is reported that H4K12 histone acetylation level is reduced in the hippocampus of old mice leading to decline in memory consolidation. H4K12 regulates the expression of several synaptic plasticity genes such as Fmn2, Myst4, Prkca etc during brain aging.

Histone acetylation and deacetylation are regulated by histone acetyltransferases (HATs) and histone deacetylases (HDACs) enzymes. We demonstrated that HDAC2 is upregulated in the hippocampus of old mice during aging and this is negatively correlated with decline in recognition memory during aging [29]. We also identified that HDAC2 mediated decrease in H3K9 and H3K14 acetylation level at the promoter region of Arc and BDNF results in deficits of recognition memory consolidation in aging as well as chemically induced amnesia [67]. Other gene manipulation studies revealed that HDAC2 negatively regulates learning and memory as its over-expression decreased dendritic spine density and spatial memory consolidation [68]. HDAC2 upregulation by H_2_O_2_ mediated oxidative stress and β amyloid peptide is also attributed to AD pathology and cognitive deficits [69].

Histone methylation has also gained importance in memory decline during aging. It is well reported that hippocampal neurogenesis decreases with age and contributes to memory deficits [70]. Doublecortin (DCX) is a cytoskeletal protein expressed by neuronal precursor cells and used as a marker for neurogenesis. It is reported that decrease in H3K4 trimethylation and increase in H3K27 trimethylation at the promoter region of DCX gene reduces its expression, eventually leading to impaired neurogenesis and memory decline in old mice [71]. These evidences clearly indicate that modifications of histones are crucial and warrant further investigations in relation to aging and neurodegeneration.

## Recovery avenues

Multiple approaches are being adopted to retard brain aging associated cognitive decline and vulnerability of disorders. These approaches including herbal interventions, lifestyle modulation, hormonal replacement therapy and stem cell based regenerative medicine eventually endeavor to modulate the molecular switches of cognitive deficits. In this regard, our laboratory has contributed on natural remedies of brain aging highlighting nootropic potential of Ashwagandha and Brahmi extracts. We reported that in animal models mimicking age related cognitive deficits, these extracts exhibit remarkable potential to enhance the expression of plasticity genes (BDNF, Arc, Neuropsin), promote neurite growth and reverse memory decline [72].

Genes altered during brain aging are also targeted specifically as recovery strategies. In AD-transgenic mice, BDNF gene delivery reversed synapse loss, normalized aberrant gene expression, improved cell signaling and restored learning and memory. Several protein kinases including protein kinase A and PI3K/AKT/mTOR pathway have been identified as crucial molecular targets in reversal of age-associated deficits in memory consolidation. Another molecular switch that is integral to brain aging related cognitive decline is the CREB dependent transcriptional machinery of memory linked genes and is considered to be a potent therapeutic target in disorders of the CNS [73].

Although these diverse avenues have proven to be beneficial, their effects are targeted and partial, and necessitate a holistic approach driving global gene expression changes. Such presumptions highlighted epigenetic modifiers as key therapeutic avenues in aging and neurodegenerative diseases. In particular, administration of different HDAC inhibitors, sodium butyrate (NaB) and Suberoylanilide hydroxamic acid (SAHA), rescued age-associated decline in H4K12 Ac and memory consolidation [74, 75]. HDAC2 selective inhibition by antisense oligonucleotides altered the histone acetylation status of synaptic plasticity genes promoter and enhanced memory in scopolamine induced amnesic mice [67]. NaB treatment increased the expression of pCREB and learning and memory in D-galactose induced aging mouse model [76]. NaB treatment also rescued cognitive impairment in APPswe/PS1dE9 transgeneic mice model of AD [77]. HDAC inhibitors have been used in different neurodegenerative and psychiatric disorders like Parkinson’s disease, Huntington’s disease, ischemic stroke, anxiety, depression, Schizophrenia etc [78]. However, the currently used HDAC inhibitors are non-selective inhibiting the activity of several HDAC enzymes and causing side effects including gastrointestinal, hematological, cardiac and metabolic dysfunctions [79]. Therefore, targeted therapy using selective HDAC inhibitors that alleviate neurological dysfunctions as well as maintain physiological balance is necessary [80]. These specific HDAC inhibitors are still to be elucidated and might come up as novel therapeutic agents in treatment of age associated neurodegenerative disorders. Besides histone acetylation, compounds altering DNA methylation have also proven beneficial in brain aging. Treatment with S-adenosylmethionine (SAM) in human neuroblastoma SK-N-SH cell cultures as well as AD transgenic mouse models silenced the expression of PS1 and Beta-secretase (BACE), reduced oxidative damage and improved cognitive performance [81,82,83,84].

**Figure 1.**
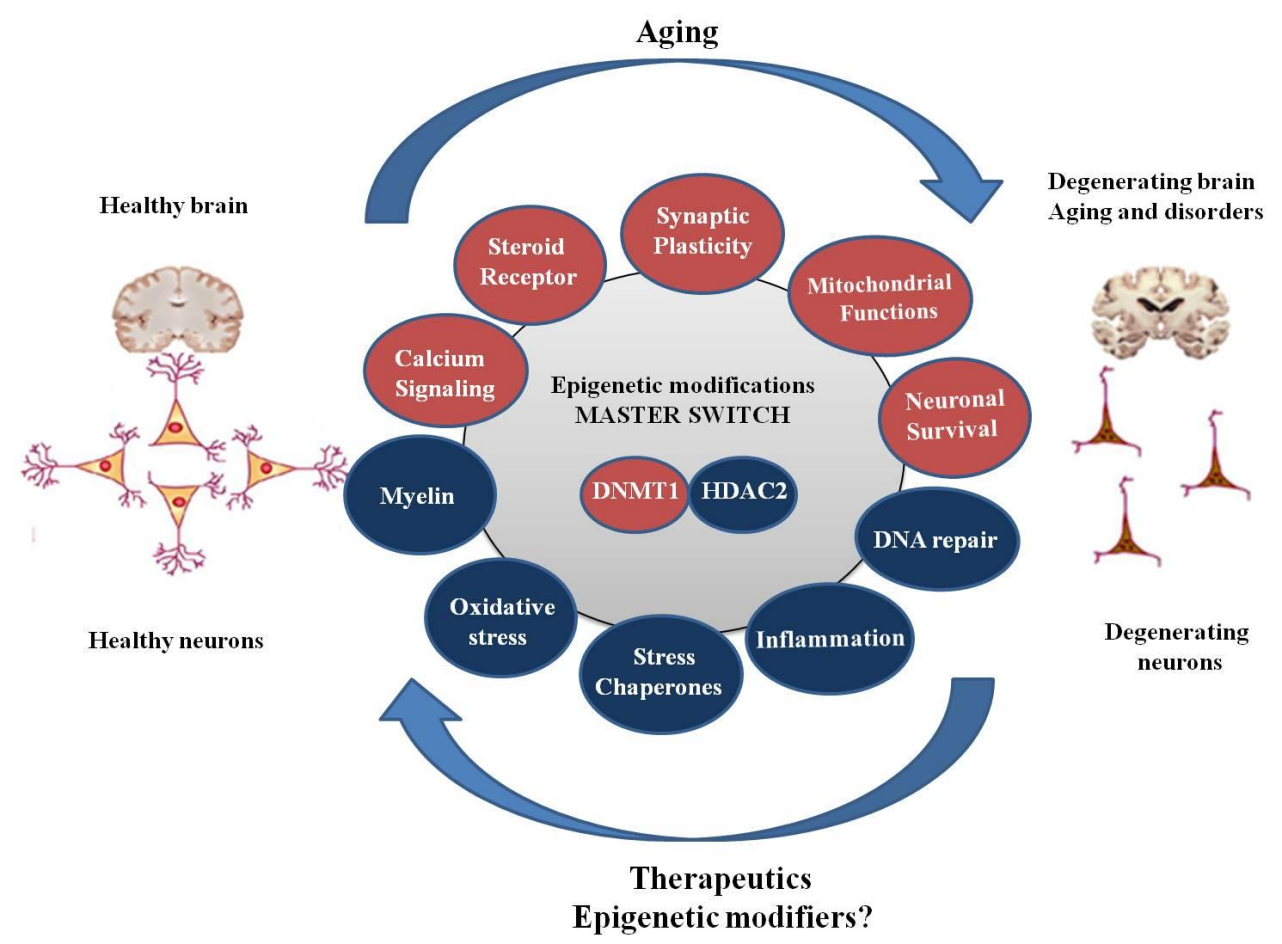
**Gene expression changes and master switch driving age related cognitive decline** Brain aging accompanies alteration in expression (red circles represent downregulation; blue circles represent upregulation) of genes belonging to multiple pathways. Epigenetic modifications particularly decrease in DNMT1 and increase in HDAC2 level might be the master regulators and accordingly epigenetic modifiers might prove ideal therapeutic targets.

## Conclusion

Age-related cognitive decline is an increasing biomedical concern. The exploration of underlying mechanisms highlighted the aberrant gene expression changes as pivotal for cognitive disabilities. However, these genes belong to multiple pathways making the recovery approach complex and arduous. Recent reports pointed towards a master molecular switch involving the epigenome driving global gene expression changes (Fig 1). In particular, chemicals or gene manipulation strategies that can promote epigenetic changes have been highlighted as holding promise for alleviating cognitive dysfunction. Further studies are warranted to investigate epigenetic marks in the regulation of cognition and reversal of age-associated cognitive decline.
